# Recent Advances in Fluorescence Lifetime Analytical Microsystems: Contact Optics and CMOS Time-Resolved Electronics

**DOI:** 10.3390/s17122800

**Published:** 2017-12-04

**Authors:** Liping Wei, Wenrong Yan, Derek Ho

**Affiliations:** Department of Materials Science and Engineering, City University of Hong Kong, Hong Kong 999077, China; weiliping1988@gmail.com (L.W.); wenroyan-f@staff.cityu.edu.hk (W.Y.)

**Keywords:** contact sensing, CMOS, fluorescence spectroscopy, time-resolved, time-gated, TCSPC, microsystems, lab-on-a-chip

## Abstract

Fluorescence spectroscopy has become a prominent research tool with wide applications in medical diagnostics and bio-imaging. However, the realization of combined high-performance, portable, and low-cost spectroscopic sensors still remains a challenge, which has limited the technique to the laboratories. A fluorescence lifetime measurement seeks to obtain the characteristic lifetime from the fluorescence decay profile. Time-correlated single photon counting (TCSPC) and time-gated techniques are two key variations of time-resolved measurements. However, commercial time-resolved analysis systems typically contain complex optics and discrete electronic components, which lead to bulkiness and a high cost. These two limitations can be significantly mitigated using contact sensing and complementary metal-oxide-semiconductor (CMOS) implementation. Contact sensing simplifies the optics, whereas CMOS technology enables on-chip, arrayed detection and signal processing, significantly reducing size and power consumption. This paper examines recent advances in contact sensing and CMOS time-resolved circuits for the realization of fully integrated fluorescence lifetime measurement microsystems. The high level of performance from recently reported prototypes suggests that the CMOS-based contact sensing microsystems are emerging as sound technologies for application-specific, low-cost, and portable time-resolved diagnostic devices.

## 1. Introduction

Fluorescence spectroscopy and microscopy have been ubiquitous research tools, serving a variety of applications ranging from biomedical diagnostics [1,2,3,4,5] to bio-imaging such as cellular [6,7,8] and molecular imaging [9,10]. In fluorescence analysis, samples are labeled using fluorescent molecules that absorb an excitation light and emit fluorescence at a longer wavelength. This fluorescence emission is subsequently detected and quantified. Steady-state and time-resolved fluorescence measurement are the two basic types of fluorescence measurements. A steady-state measurement is performed by exciting the fluorescent labeled sample with a beam of continuous light and recording the emission spectrum. Time-resolved fluorescence measurement is a technique where the fluorescence decay profile is recorded with high temporal resolution after a pulsed excitation. Since steady-state observation is the average of the time-resolved phenomena, much of the information that characterizes the fluorescent molecular interactions and the surrounding chemical environment is lost [11]. Compared to steady-state fluorescence measurement, time-resolved fluorescence measurement provides superior sensitivity and selectivity in many analytical applications. The measurement where the fluorescence lifetime is extracted from the fluorescence decay profile is fluorescence lifetime measurement (FLM) [12].

Two types of FLM techniques are prominent, namely time-gated [13] and time-correlated single photon counting (TCSPC) [14]. The time-gated method measures a fluorescence decay curve either using several time gates with equal width or by time gate scanning. In the former method, lifetimes shorter than hundreds of picoseconds are typically difficult to resolve [13]. The time gate scanning method achieves higher temporal resolution at the expense of photon collection efficiency, as the narrow gate excludes the majority of the detected photons [15]. At present, most of the FLMs are performed using TCSPC, which achieves both high temporal resolution and high optical efficiency [16]. In commercially available TCSPC instruments, the temporal resolution, which depends on the time bin width, is typically as low as hundreds of femtoseconds. With a high-frequency excitation source, acquisition time as short as several milliseconds can be achieved with these commercial equipment [17].

A wide variety of FLM instruments are commercially available. However, they are typically bulky and expensive, targeting bench-top, laboratory-based applications [18,19]. This is due to two key technological limitations in both the electronic and optical domains. First, the high pico-to-nanosecond temporal resolution requires complex signal processing electronics. Discrete electronic components render highly parallel, highly integrated (e.g., greater than one million transistors) implementations difficult, which limits device throughput. Second, an elaborate optical path involving a system of lenses is often applied to focus the extremely low intensity fluorescence emission light onto the photon detector as well as the laser into a very small plane for scanning imaging. Existing FLM systems are typically not suitable for ubiquitous utility, for example, in point-of-care and implantable applications [20], where low cost and high portability are required.

To minimize system size, cost, and improve throughput, the aforementioned limitations must be overcome. In terms of electronics, reduction of complexity, size, and power consumption is achieved through implementation utilizing the CMOS technology. Nanometer-scaled CMOS technology allows large photon detection array and signal processing electronics to be fabricated on the same chip, which enables array implementation with high parallelism [21]. In terms of optics, to eliminate the need for complex and bulky optical elements, contact sensing has been extensively studied in recent years. Contact sensing is a method in which samples are placed in close proximity to the sensor surface without intermediate optics. Due to the short distance between the sensor and sample, the optical loss can be small. Optically efficient contact sensing systems have been demonstrated covering widespread applications, such as cell manipulation process [22], high-resolution imaging [23], and detecting cellular functions at the single-cell level [24]. 

In this paper, we present a study of recent advances in CMOS fluorescence lifetime analytic microsystems, in which the combination of contact sensing and time-resolved circuits are expected to play a key role on the roadmap of application-specific, low-cost, and portable diagnostic devices. Section 2 describes the principle of FLM, including time-gated and TCSPC techniques. Section 3 reviews the advanced CMOS FLM microsystems. Section 4 reviews CMOS contact steady-state and time-gated microsystems. Section 5 explores the feasibility of contact TCSPC systems implemented in the CMOS technology. Section 6 concludes the paper.

## 2. FLM Techniques

### 2.1. Time-Gating

**Figure 1:**
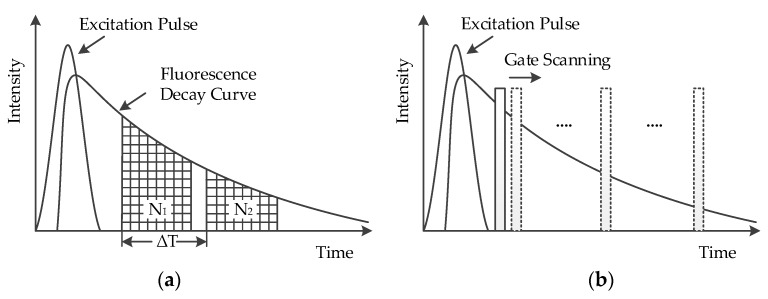
The two methods of time-gated fluorescence detection. (**a**) The fluorescence decay curve detected using two time gates with equal width; (**b**) the time gate scanning method.

Time-gated fluorescence is a prevalent time-resolved fluorescence technique where a pulsed excitation source is used to illuminate the sample. The time-gated method measures a fluorescence decay curve either within several time gates with equal widths or by time gate scanning. In the former method, to measure mono-exponential fluorescence decay, two time gates with equal width are typically enough, whereas for multi-exponential decay more time gates are required [25]. The fluorescence lifetime with a mono-exponential decay is calculated from Equation (1) [26].

(1) $$\tau =\frac{\Delta T}{\ln\left(N_{1}/N_{2}\right)}$$

where Δ*T* is the time interval between two time gates with equal width, *N*_1_ and *N*_2_ are the photon numbers within the time gates, respectively, as shown in Figure 1a. Empirically, when the time gate width Δ*T* is equal to 2.5 τ, the error between the calculated and the theoretical lifetime is the smallest. Since the measured fluorescence decay is the convolution of the instrument response function (IRF) with the theoretical fluorescence decay of the equipment, the signal becomes exponential after the IRF drops to a negligible level. Therefore, the photons detected in the first part are not counted. For measuring lifetimes below 500 ps, this typically leads to a very low efficiency [27].

Alternatively, in the time gate scanning method, photons are detected in a narrow time gate of tens of picoseconds synchronized with the excitation pulse. After repeating sufficient times, the time gate shifts automatically by a picosecond time step and detection is repeated, as shown in Figure 1b. At the end of the measurement, a histogram is constructed [12]. However, since time gating rejects the majority of the detected photons, detection efficiency is compromised.

### 2.2. TCSPC

**Figure 2:**
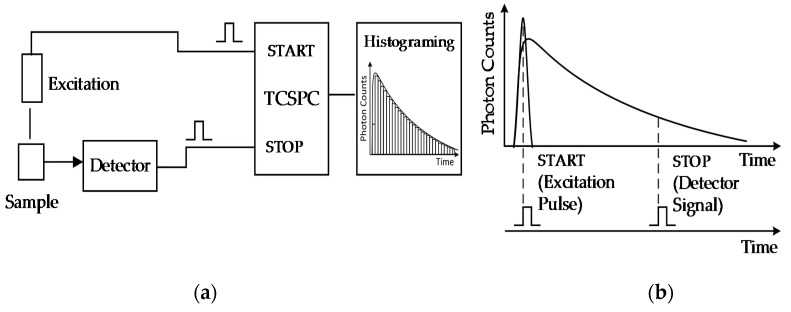
Time-correlated single photon counting (TCSPC) operation principle. (**a**) System schematic; (**b**) timing diagram of the start-stop watch mode [29].

TCSPC has been the most widely used time-resolved fluorescence technique due to its high optical efficiency and temporal resolution. A typical TCSPC apparatus consists of a pulsed excitation source, a single photon detector, a timing electronics, and a computer to extract the fluorescence lifetime, as depicted in Figure 2a. The detector is typically a PMT for a conventional system and a single photon avalanche detector (SPAD) for a system with CMOS electronics. The timing electronics typically consists of a time-to-digital converter (TDC), or a time-to-amplitude converter (TAC) combined with an analog-to-digital converter (ADC). In general, TAC achieves higher resolution and ADC linearity, but suffers limited temporal measurement range and operating temperature range. TDCs are more compatible with CMOS implementation and less sensitive to external disturbances. Therefore, TDC has garnered an increasing amount of interest in recent years [28]. When the fluorophore is excited, a synchronization pulse from the excitation source driver is delivered to the START input of the timing electronics. After the excitation is turned off, the fluorophores in the sample emit fluorescence, which is detected by the single photon detector. Once a photon is detected, the detector delivers a pulse to the STOP input of the timing electronics. The time interval between the START and STOP pulse is the photon time-of-arrival and this timing mode is called “start–stop watch” mode, shown as Figure 2b. After a large amount of excitation–detection cycles, a histogram depicting the fluorescence decay is obtained by attributing the arrival times of photons to the corresponding time bins. 

The single photon pulse from the detector is a sequence that captures the photon arrival statistics, which is a Poisson process. Some signal periods contain one photon, whereas many signal periods record no photons. The excitation intensity is typically moderated so that multiple photons arriving during the same period is rare, which is necessary to prevent a form of distortion known as photon pile-up. The pile-up effect is due to the fact that subsequent photons within the same period cannot be detected, which results from the dead time incurred by the detection of the first photon. 

## 3. CMOS Implementation of FLM

CMOS has become a prominent technology for implementation on very-large-scale integration (VLSI) chips. CMOS circuits allow a high density of logic functions integrated on a single chip and a high processing speed. Benefitting from a high level of integration, CMOS implementation has shown great potential in parallel processing [30,31], high noise immunity, and low power consumption [32,33]. With these advantages, highly integrated, low-cost, and low-power consumption FLM systems are achieved by fabricating a large number of single photon detectors and signal processing circuits on the same chip. Therefore, CMOS FLM systems are experiencing breakthroughs in integration level, pixel area, array size, time resolution, and power consumption. The main parameters in recent state-of-the-art CMOS FLM systems are listed in Table 1.

In the pursuit of higher temporal resolution and increased throughput, FLM systems need to be designed with a higher level of integration and parallelism. Chip-level, column-level, and pixel-level are three integration levels of the FLM microsystems. 

On the chip-level, the SPAD and TDC are both fabricated on the same chip whereas they are in separate block and the TDCs process the data from the SPADs sequentially. A fluorescence lifetime imager integrates the on-chip TDC with a 64 × 64 SPAD array is proposed in [45]. In this design, the array of detector pixels, the timing generation circuitry, and a set of latches are the primary blocks of the proposed fluorescence lifetime imager. The latches at the output of each column is utilized to store the timing information and output state of each pixel in this column. In another design, 1024 circular SPADs with an 8 μm diameter active area arranged in a 32 × 32 array and an 8-channel TDC arranged in a 2 × 4 array are integrated on a chip-level to achieve single photon counting [16]. In this system, for each excitation period, the core architecture for recording the photon arrival time is formed by eight TDC pairs. This novel design can greatly avoid the pile-up distortion. However, for TCSPC applications, the probability of detecting a photon per excitation period is about 1%. Therefore, TCSPC microsystems on a chip-level integration suffer a relatively long acquisition time. 

On the column level, a column of SPAD pixels, or several of them, sharing a TDC. This enables all pixels to detect photons while reducing the space necessary for TDCs. An efficient sharing of the TDC with eight SPAD pixels is presented in [48]. A 1 × 400 array of backside-illuminated SPADs for photon detection is in the top tier substrate while the time-to-digital conversion is in the bottom tier. Upon this two-tier design, the area of the whole microsystem is greatly reduced. However, if more than one photons arrive during the same excitation period, only one of them is counted. This level of TDC sharing may cause a slightly distortion of decay if the light intensity is not low enough.

On the pixel level, TDCs are integrated in each pixel which means that each pixel corresponds to specific photon timing and counting circuits to achieve high data rate processing through parallelism. A 16 × 4 array of SPADs with in-pixel photon counting circuits in the time-gated method are implemented with 0.35 μm high-voltage CMOS technology [34,35]. Owing to the highly parallel processing, a 32 × 32 array of low dark count SPADs with in-pixel TDC achieves imaging with a frame rate over 50 fps [37]. A 512 × 128 SPAD array with in-pixel TDC enables a fluorescence lifetime imaging frame rate as high as 156 kfps [40]. The disadvantage of the pixel-level integration is that the fill factor of the imager is dramatically reduced and consequently reduces the photon detection efficiency, rendering the imager unsuitable for applications with very low light intensity. To improve the fill factor of SPADs, 3-D integration technology, in which the photon counting circuits are fabricated on a separate silicon wafer, has been proposed [52,53]. The SPAD array fabricated in the top silicon layer of a silicon-on-insulator (SOI) wafer is flip-chip bonded to the photon counting circuits wafer. After etching the silicon substrate of the SOI wafer, the SPADs could be illuminated from the backside through the buried oxide layer.

In the context of the fill factor, the draining-only modulation (DOM) lock-in pixels based on the time-gated method have a simple pixel structure, thereby achieving a spatial resolution higher than that of SPADs [50]. However, it suffers a relatively low transfer speed and that parallel output is difficult to realize. Another time-gated two-tap lock-in pixel using lateral electric field charge modulation (LEFM) and two-stage charge transfer techniques is proposed in [51]. The sensor achieves a high time resolution by the LEFM technique and obtains a low temporal noise by true correlated double sampling (CDS) operation, and a high spatial resolution because of the compact pixel configuration.

As depicted in Table 1, with higher integration level and larger array size, the power consumption of the microsystems is higher. The power consumption in chip-level systems is a little higher than that in column-level because they have a larger array size. Systems implemented on the pixel-level, i.e., pixel-parallel implementations, normally suffer from high power consumption. The largest array size is achieved by the time-gated method, whereas the other best performances are realized by the TCSPC method.

Although there is always a tradeoff among integration level, array size, and power consumption, the development of CMOS FLM microsystems has already reached a relatively mature stage. Different types of systems can be fabricated according to their own application requirements.

## 4. Recent Advances in Contact Sensing

**Figure 3:**
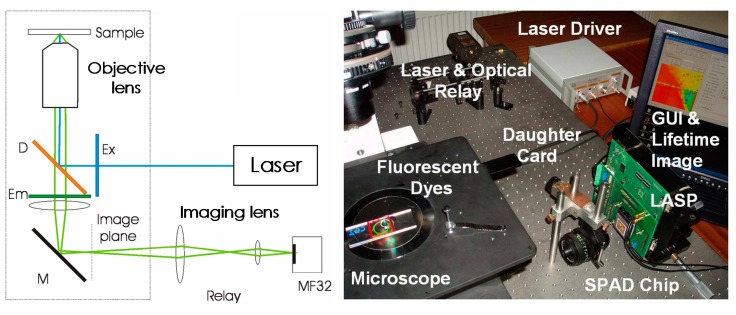
Schematic and photo of a conventional optical system of time-resolved fluorescence system (MF32). LASP is the motherboard of the SPAD array daughterboard. © (2010) Optical Society of America. Adapted, with permission, from [57].

For fluorescence measurement, the fluorescent emission is typically orders of magnitude weaker than the excitation light. Without sufficient filtering to block the excitation and lens to focus the emission, a high background noise caused by the detected excitation photons typically distort the decay. Typical optical setups for wide-field fluorescence measurements consist of objective lens, imaging lens, excitation filter, dichroic mirror, and emission filter. CMOS fluorescence imaging sensors are commonly tested with a microscope [46,47,51,54,55,56], as shown in Figure 3. 

In the optical path, both the objective lens and imaging lens are utilized to focus the light thus avoiding stray light. Excitation filter is typically a bandpass or short-pass filter that passes only the fluorophore absorption wavelengths while rejecting other (parasitic) wavelengths that are generated by the source. These parasitic wavelengths, if not filtered, can often be picked up by the detector, resulting in undesirable background noise. The excitation filter can also be a neutral density (ND) filter for a single-wavelength excitation source. The dichroic mirror spectrally separates excitation and emission light by transmitting and reflecting light as a function of wavelength. Therefore, at the dichroic surface, the excitation light is reflected to stimulate the fluorescent sample, while emission light transmits. The emission filter is typically a long-pass filter that transmits emission light with longer wavelengths and rejects excitation light with shorter wavelength. To further prevent the excitation reaching the imager, a mirror is used to reflect the emission light reaching the CMOS imager perpendicular to the excitation light. With the optical system, a high level of excitation rejection can be achieved due to the spectral and spatial separation. However, to settle the complex optical path, it requires a large space, which renders the whole system bulky. For conventional imaging systems, the ultimate limitation of the spatial resolution is the diffraction of light, which is dependent on the wavelength and the numerical aperture. For contact imaging systems, the spatial resolution is a function of both the pixel size and the distance between the sample and the pixels [58]. Although current contact imaging systems cannot typically rival the spatial resolution of conventional systems, they can circumvent the bulky and complex optical path and maintain the ability of imaging fluorescent samples at the micro level. Many fluorescent lab-on-a-chip (LoC) devices and systems performing imaging have been demonstrated in [59,60,61]. In the following subsection, state-of-the-art CMOS fluorescence detection and imaging systems using contact sensing are surveyed.

### 4.1. Steady-State Contact Sensing 

In contact sensing systems, the samples are very close to the sensor surface and no lens is required to focus the light. Therefore, the main design challenges are introducing the sample in close proximity above the sensor and providing adequate excitation rejection (giving the space constraint between the sample and the sensor). For most contact fluorescence sensing systems, a polydimethylsiloxane (PDMS) microfluidic chip, due to its transparency and tractability, is commonly used for the transport and storage of fluorescent samples. The transparent PDMS provides a hermetic seal to silicon dioxide and allows for the transmission of the excitation light in the visible and near-ultraviolet wavelength range. A multi-layer thin-film filter with a total thickness that is hundreds of micrometers is coated or placed directly onto the sensor to reject excitation light while transmitting the emission light. 

An approach to miniaturizing the fluorescence measurement system by combining PDMS microfluidics with a filtered CMOS imager is presented in [59]. The thin-film filter is formed by the depositing alternate λ/4 layers of silicon dioxide and silicon nitride. This multi-layer thin-film filter has a high transmittance at the fluorescence emission wavelength range while providing a strong rejection of the excitation wavelength. The PDMS microfluidic chip, including 11 channels that are 100 μm wide and 14 μm deep, and spaced 100 μm apart, is placed directly onto the thin-film filter so that it is close to the fluorescence imager. This class of microfluidic integrated system is suitable for high-throughput biological drug delivery and analysis. One disposable microfluidic chip, with a 1 μm-thick silicon nitride membrane, is assembled on the interference filter coating on the surface of a CMOS color imager with 5 mega pixels [60]. This system can be used to measure the concentration of fluorescent samples and imaging fluorescent mammalian cells with a 15–20 μm diameter. A handheld fluorimeter imager with an integrated PDMS well and long-pass optical filter is presented in [61]. The polymer film filter with high rejection chromophore embedded is coated on the fluorescence imager. These handheld bioassay instruments can detect the metabolic activity and viability of cells, which can be utilized for cytotoxicity and pathogen measurements. 

**Figure 4:**
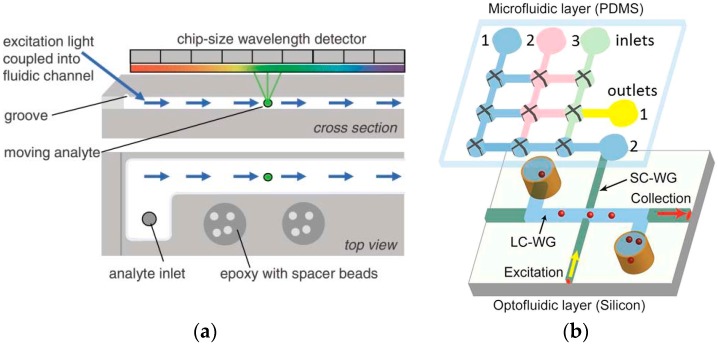
(**a**) The cross section and top view of the fluidic channel. The excitation light is coupled into the fluidic channel and the imager records the fluorescence emission of the analytes while they move along the channel. Reproduced from [63] with permission from The Royal Society of Chemistry. (**b**) The prototype of the ARROW chip for sample preparation and single nucleic acid measurement. Reproduced from [64] with permission from The Creative Commons Attribution 4.0 International License.

The microfluidic channel can also be used to guide the excitation light other than sustaining the fluorescent sample, meaning it acts as optical waveguide. Since the refractive index of the liquid core is lower than the channel walls, the light, based on the principle of anti-resonant reflection optical waveguide (ARROW), can only propagate in the channel rather than the channel walls [62]. ARROW enables an extended interaction length, thereby improving the interaction efficiency between excitation light and analytes. A chip-size fluorescence spectrometer with an ARROW fluidic platform and a linear variable band-pass filter integrated on a CMOS imager for analyzing moving objects is proposed in [63]. The prototype of the chip-sized spectrometer is shown in Figure 4a. The liquid waveguide structure is defined on an acrylic sheet and a glass substrate with four glass beads sandwiched between them. The fluorescence dyes in the fluid are then continuously excited by the excitation light that propagates through the liquid waveguide. Through using multiple fluorescent dyes to identify biological molecules, this device enables point-of-care, real-time detection, and analysis of pathogens. Based on the micro-electromechanical systems (MEMS) fabrication, ARROWs can be easily integrated on-chip. An ARROW structure guiding emission intersects with a solid-core waveguide for guiding excitation, as shown in Figure 4b [64]. 

A contact measurement system with higher performance of thin-film filter permits higher measurement sensitivity of fluorophores. A thin-film filter with optical density (OD) above 6.0 within the desirable wavelength range integrated on a 128 × 128 fluorescence imager array is presented in [65]. The emission intensity of the cyanine-3 fluorophore is measured as a function of fluorophore concentration and the estimated measurement sensitivity is as high as 5000 fluorophore/μm^2^.

The thickness of the thin-film filter is smaller than the wavelength, which set a strict requirement on fabrication. With a special design of the CMOS imager, the emission filter can be non-essential for a fluorescent measurement microsystem, thereby further shortening the distance between the sample and the imager. An on-chip filter-less fluorescence imager is demonstrated to successfully detect the fluorescent sample and is able to detect fluorescence intensity of approximately 1/300 of the excitation [66]. According to the principle of the wavelength-dependent optical absorption coefficient, the optical intensity of a specific wavelength can be obtained based on the penetration depths in silicon. A spectral-multiplexed filter-less fluorescence contact imager with a microfluidic chip for deoxyribonucleic acid (DNA) detection is proposed in [67]. The filter-less CMOS microsystem utilizes the polysilicon gate as a band-pass filter. The spectral-multiplexing capability of the microsystem has been validated in the detection of DNA targets with two colors (e.g., red and green) of CdSeS/ZnS quantum dots labels, simultaneously. The detection limit at a sample volume of 10 μL for two targets is 240 nM and 210 nM, respectively. 

**Figure 5:**
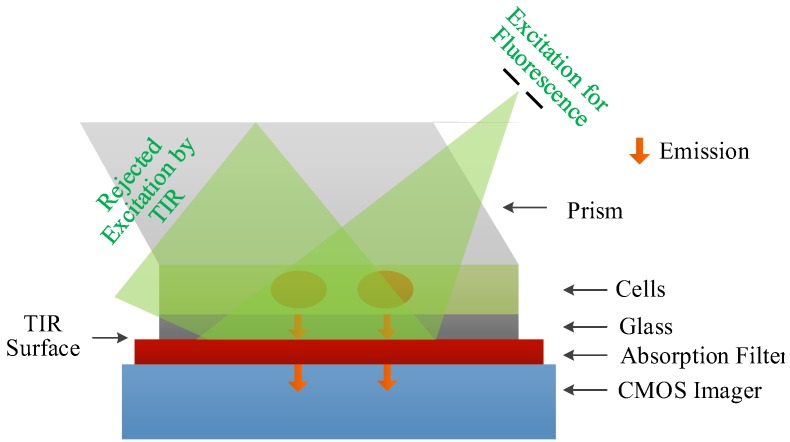
On-chip lens-free holographic fluorescent imaging platform with a wide field-of-view of 2.5 cm × 3.5 cm. Adapted from [24] with permission from The Royal Society of Chemistry.

An appropriate way to introduce the excitation light can improve the excitation rejection capability of the contact microsystem. One approach to excitation rejection is utilizing miniaturized optics to guide excitation away based on the principle of total internal reflection (TIR). An on-chip wide-field holographic fluorescent imaging platform without any lenses and mechanical scanners is presented in [24], as shown in Figure 5. Lens-less imaging is achieved by reflecting the excitation beam at the bottom of the sample container. The incident angle of the excitation beam is adjusted by the prism. To eliminate the scattering excitation at the bottom facet of the prism, the index matching gel is applied at the interface of the prism and the glass. The emission light does not obey TIR, thereby directly reaching the entire field-of-view of the imager-array. Such a wide field-of-view contact fluorescent imaging platform enables highly efficient detection and high-throughput screening of rare cells.

Typically, the excitation source of a fluorescence measurement system is a standalone module. To achieve a highly integrated fluorescence detection system and be potential for implantable applications, the excitation source needs to be integrated on-chip [68]. Such a highly integrated and implantable fluorescence imaging system can be utilized to observe various neural activities of the mouse brain in a freely moving state with high-sensitivity. The excitation is rejected by a combination of dedicated thin-film filters. A weak green fluorescence emitted by green fluorescence protein (GFP) is observed in positive cells from mouse brain slices [20].

### 4.2. Time-Gated Contact Sensing 

In FLMs, the excitation source is pulsed; and, if the pulse width is narrow enough, the detected photons are actually the emission photons. However, the realizable pulse width of the excitation is often wider than desired. Therefore, a filter is still needed to reject the excitation light that can reach the detector, although emission filters are not essential for FLMs in theory. Therefore, compared to the steady-state measurement where the excitation is rejected spatially and spectrally, FLMs have greater excitation rejection ability because the excitation light can be rejected temporally other than spatially and spectrally. For the time-gated method, only the photons in the detected time window are detected. By properly designing the width and location of the time windows, the majority of excitation photons can be rejected. 

With simplified optics, the samples can be placed close to the detector surface to achieve contact sensing, via micro-capillary, microfluidic channel, micro-reservoir, and so on. The following is a survey of CMOS time-gated systems demonstrating different variations of contact sensing.

A contact time-gated fluorescence system utilizing micro-glass capillary to hold the fluorophore solution is presented in [69]. The glass capillary with an internal diameter of 550 μm and full of fluorescent solution [70] is suspended close to the photon detector array surface. The pulsed excitation beam excites the fluorescent samples from a direction perpendicular to the capillary. The SPAD array with each pixel occupying a 180 × 150 μm^2^ area is used to detect the emission light. This time-gated fluorescence measurement system has been successfully demonstrated to detect the fluorescence emission without the aid of any focusing optics or optical filters. The average value of the measured lifetime of this system is 19.54 ns, which is in accordance with the lifetime of 19.97 ns measured by a commercial TCSPC system. This contact time-gated microsystem shows that the excitation photons are almost eliminated by the time gate. 

An on-chip, filter-less all-digital time-gated CMOS fluorescence imager for DNA detection is presented in [71]. A layer of PDMS is coated on the chip surface to sustain the fluorescent samples as well as to avoid short circuits. Three 200 nL volume spots are fabricated on the surface of the PDMS in which three different concentrations—36 μM, 18 μM, and 9 μM—of Cy5-linked oligonucleotide (30 mer) are dropped for detection. Based on the time-gated method, the excitation is eliminated by appropriately selecting the width and position of the time gate, and the weak emission is then observed. This filter-less system achieves a detection limit of DNA of 14.6 μM (8.8 × 10^8^ molecules per pixel) [71]. 

An angle-sensitive SPAD array able to conduct lens-less 3-D fluorescent lifetime imaging is presented in [72,73]. By the diffraction gratings, the incident angle of light is extracted and 3-D localization of the fluorescent sources at a micrometer scale is enabled. The fluorescent sources are distinguished based on their lifetimes obtained by the time-gated method. 

A time-gated fluorescence contact detection microsystem with probes immobilized directly on the sensor surface is presented in [21]. Since the laser diode is right above the detector array, a 23-layer SiO_2_/TiO_2_ thin-film filter with OD 5 is still utilized to achieve high background rejection. 

To maximize the photon collection efficiency, the analytes should be placed in immediate proximity to the sensor surface [74]. For DNA detection, the DNA probes can be immobilized on the surface of the sensor by contact-pin spotting [75] or covalent attachment techniques [76]. To aid a good attachment, the surface of the sensor should be firstly cleaned and epoxy-derivatized with 3-glycidoxypropyltrimethoxysilane. The sensitivity of this time-gated sensor is as low as several 10^8^ photons per square centimeter on average [76].

**Figure 6:**
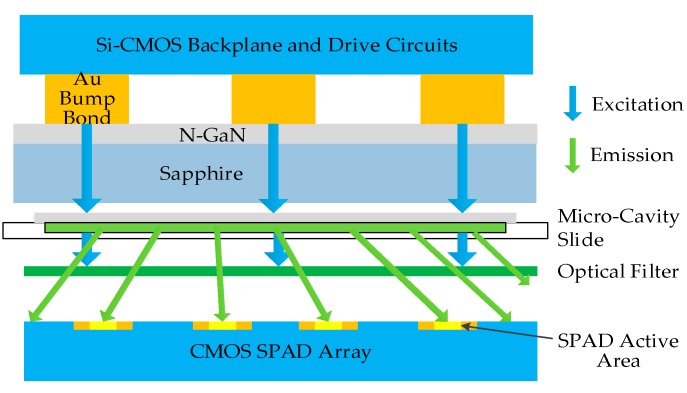
The cross section of the integrated time-gated fluorescence microsystem. The fluorescent sample is sandwiched between a micro-cavity slide and a cover slip. The long-pass filter eliminates the excitation light. © (2010) IEEE. Adapted, with permission, from [34].

A highly integrated and portable time-gated fluorescence system combining excitation, filtering, and detection is described in [34], as shown in Figure 6. The excitation is an 8 × 8 array of AlInGaN blue micro-pixelated LEDs with an array of drivers implemented by CMOS technology. The narrow pulse width of 777 ps enables a short lifetime resolution. The shortest lifetime measured in this work is 1.3 ns. The fluorescent sample is sustained in a micro-cavity slide and sealed by a cover slip, and these are placed between the LED chip and the detection chip. The emission light is then detected by the detector array opposite the LED chip. A 514 nm long-pass filter is chosen to further distinguish the emission from the 450 nm excitation. By eliminating the PMTs, bulk optics, and discrete electronics, this design has already achieved a high level of system integration. In order to further reduce the device dimensions, the optical filter is intended to be removed and the microfluidics is to be incorporated to deliver the sample.

## 5. Perspective

The TCSPC method has higher photon collection efficiency than the time-gated method because without any time gates all detected photons contribute to the measurement. However, the TCSPC method suffers worse background rejection performance relative to the time-gated method, thereby relying more on the performance of the emission filter. Therefore, the contact TCSPC system is not as common as the contact time-gated system, although the CMOS TCSPC microsystems are more pervasive than CMOS time-gated microsystems. In fact, two preliminary prototypes have been demonstrated in [44,77] and have shown the feasibility of CMOS contact TCSPC microsystems.

The first prototype is an 64 × 64 active microarray platform implemented in the CMOS technology for time-resolved Förster resonance energy transfer (TR-FRET) oligonucleotide assays [77]. The probes are firstly immobilized on the substrate and labeled with a donor fluorophore. The target analytes are labeled with an acceptor fluorophore. The extent of hybridization of probe and target analyte is then estimated by the changes of the lifetime of the donor. The 22-layer TiO_2_/SiO_2_ thin-film long-pass filter deposited on the surface of the microarrays achieves a 30–40 dB rejection at the excitation wavelength and 92% transmission at the emission wavelength. The detection limit of the target analyte concentration is 20 nM. 

The second prototype is a pixel-level integrated CMOS array for high-speed fluorescence lifetime imaging [44]. The performance of this imager is demonstrated by directly placing a dish of fluorescein dyes over the detector array. A ceramic cover is used to cover a portion of the SPAD array to define the imaging pattern. A 550 nm emission filter is employed to reject the excitation light.

In terms of minimizing the requirement of the high performance thin-film filter, utilizing a very short excitation pulse and applying some optical waveguide structures as depicted in [78] to separate the excitation and emission are also useful methods.

Combining the advantages of contact sensing and CMOS technology, the CMOS contact TCSPC sensor demonstrates superior performance both temporally and spatially. Since this unique combination for TCSPC has barely been studied, as only the above two key examples can be given, the development of high-performance, high-throughput, low-power, low-cost, and portable TCSPC sensors is a promising direction of future work.

## 6. Conclusions

TCSPC is the most pervasive time-resolved fluorescence measurement technique for its high temporal resolution and photon collection efficiency. Well supported by contact sensing and the CMOS technology, the CMOS contact TCSPC system has been shown to have a high level of integration, low power consumption, high throughput, a small size, and reduced cost. Based on ample demonstrations of steady-state and time-gated systems combining contact sensing and the CMOS technology, CMOS contact TCSPC microsystems are expected to enable high-frame-rate fluorescence lifetime imaging and emerging applications, particularly in the portable domain.

## Figures and Tables

**Table 1:** Main parameters of several CMOS FLM microsystems including their single photon detector and photon counting module. The best merits for each option are in bold. TG stands for time-gated.

Detector Type	Pixel Area ^1^	Array Size	Total Area ^2^	Time Resolution ^4^	Power Consumption	Integration Level	Technique	Refs
SPAD	200 × 200	16 × 4	3.18 × 3.1	80 ps ^4a^	Nil	Pixel-level	TG	[34,35]
SPAD	50 × 50	32 × 32	Nil	50 ps	Nil	Pixel-level	TCSPC	[36]
SPAD	Nil	32 × 32	Nil	54 ps	Nil	Pixel-level	TCSPC	[37]
SPAD	**Φ 6 μm ^3^**	128 × 1	3.12 × 1.1	21.4–**8.9 ps**	Nil	Pixel-level	TCSPC	[38]
SiPM	Φ 8 μm	32 × 32	1. 3 × 1.7	50 ps	9.5 mW	Chip-level	TCSPC	[16]
SPAD	250 × 250	1 × 1	0.3	10 ps	<80 mW	Pixel-level	TCSPC	[39]
SPAD	25 × 25	32 × 32	Nil	~ns ^4b^	Nil	Pixel-level	TG	[15]
SPAD	24 × 24	512 × 128	13.5 × 3.5	4 ns ^4b^	1.65 W	Pixel-level	TG	[40]
SPAD	Φ 30 μm	64 × 32	9.6 × 4.8	120 ps ^4b^	50 mW	Pixel-level	TG	[41,42]
SPAD	Φ 30 μm	32 × 32	9 × 9	312 ps	430 mW	Pixel-level	TCSPC	[43]
SPAD	48 × 48	64 × 64	Nil	62.5 ps	8.79 W	Pixel-level	TCSPC	[44]
SPAD	40 × 40	64 × 64	4 × 4	350ps	1.4 W	Chip-level	TCSPC and TG	[45]
SPAD	Nil	1024 × 8	24.7 × 0. 8	250 ps	Nil	-	TG	[46]
SPAD	23.8 × 100	256 × 2	**6.61 × 0.958**	320 ps	Nil	Pixel-level	TCSPC	[47]
SPAD	64 × 47	1 × 400	0.77 × 5	49.7 ps	**7 mW**	Column-level	TCSPC	[48]
SPAD	Φ 100 μm	60 × 1	9.3 × 2	250 ps	Nil	Pixel-level	TCSPC	[1]
Lock-in pixel	7.5 × 7.5	256 × 256	Nil	250 ps ^4b^	Nil	Pixel-level	TG	[49]
Lock-in pixel (DOM)	7.5 × 7.5	**256 × 256**	Nil	~ns	Nil	Pixel-level	TG	[50]
Lock-in pixel (LEFM)	11.2 × 5.6	256 × 512	7 × 9.3	10 ps ^4c^	540 mW	Pixel-level	TG	[51]

^1^ The unit of pixel area is in μm^2^. ^2^ The unit of total area is in mm^2^. ^3^ This is the diameter of each pixel. ^4^ For the TCSPC microsystems cited here, the time resolution represents the time bin width of the TDC or the least significant bit. For time-gated microsystems, the definition of the time resolution varies. ^4a^ This is the estimated measurement error, based on SPAD rms jitter. ^4b^ This is the time gate width. ^4c^ This is the standard deviation of the fluorescence decay profile.
